# How Can We Increase Neighbors' Intention to Report Intimate Partner Violence Against Women During the Pandemic?

**DOI:** 10.1177/10778012211034203

**Published:** 2021-10-21

**Authors:** Ainara Nardi-Rodríguez, Nerea Paredes-López

**Affiliations:** 1 Science Park Company, Miguel Hernández University, Centro Iguala, Alicante, Elche, Spain

**Keywords:** intimate partner violence against women, bystander behavior, neighbors, reasoned action approach, media campaigns

## Abstract

During the pandemic, neighbors can be potential allies to prevent intimate partner violence against women (IPVAW). Based on the reasoned action approach, we identified the predictors and the most relevant beliefs behind neighbors' intention to report to authorities that a woman is being victimized by IPVAW. A total of 352 Spanish participants completed a questionnaire. The regression analysis showed that perceived control and subjective norm were the best predictors of the intention (33% explained variance). Social media campaigns should target perceived inhibitors such as citizens' guilt for reporting ambiguous cases and close referents (friends and family) as prescribers of the helping behavior.

## Introduction

Intimate partner violence is a health and social issue that overwhelmingly affects women worldwide, who incomparably suffer the greatest burden of intimate partner homicide (82%) (United Nations Office on Drugs and Crime [UNODC], 2019; van Gelder et al., 2020).

Although official reports are scarce, worldwide institutions and women's rights advocates have pointed out the alarming increase in the number of reports and support requests to helplines and assistance services during the COVID-19 pandemic (see Boserup et al., 2020; Bradbury and Isham, 2020; Un Women, 2020). For instance, the New York City Police Department received 10% more reports of domestic violence in March 2020 in comparison with the same month the previous year (Boserup et al., 2020). In New South Wales (Australia), during the COVID-19 eruption, 40% of frontline workers received an increase in requests for help (UN Women, 2020). RESPECT, the U.K. national domestic violence charity, had to deal with 97% more calls from 16 March to 19 April 2020, along with 185% more emails, and their webpage has been visited 581% more (United Nations, 2020). In Spain, in only 2 months (April and May 2020), there was an increase of 61.5% in the request of assistance services compared within the same period of the previous year (Percharromán, 2020). Furthermore, almost 9,000 aggressors have been arrested by the National Police Department during the 3-month state of emergency period (El País, 2020). Thus, women and children are pushed to live one of the worst nightmares: a state of lockdown with their aggressors with no outside contact or very limited access to support services (Campbell, 2020). Particularly in this difficult situation, neighbors can be active social agents in the detection and reporting of suspicious cases (Banyard et al., 2019; Campbell, 2020; United Nations, 2020).

Experts insist that public media needs to target citizens to engage them in helping end intimate partner violence against women (IPVAW) (van Gelder et al., 2020). In Europe, it is estimated that 17% of the population knows a woman in their neighborhood that has suffered or suffers from IPVAW (European Commission, 2016). In Spain, this number rises up to 19% (Government Delegation for Gender-based Violence [GDGV], 2014). However, in 2020, less than 4% of the calls received by the main hotline for IPVAW support and emergencies were made by “others” or anonymous callers, which we infer includes neighbors (GDGV, n.d.). A Spanish study estimated that up to 60.5% of IPVAW victims suffer from violence every day and 32% weekly (Fernández-González et al., 2017). As a result, some neighbors should be aware of the fact that there might be cases of IPVAW happening next door. This has become more evident during this pandemic as the number of IPVAW cases has increased and people are advised to spend more time at home.

Helping behaviors have been a target of research for decades. Experts highlight multiple personal, situational, and community factors behind helping behaviors. For instance, Penner and Orom (2010) identified a prosocial personality orientation. At a contextual level, Latané and Darley (1970) considered that helping behaviors are a result of a cognitive process that is subjected to different situational variables. That is to say, the more ambiguous a situation is, the less likely people are to help. The presence of other witnesses during an aggression (the “bystander effect”) can lead to the pluralistic ignorance effect, whereby bystanders assumie the group's behavior is the norm (i.e., not acting) even if it is against their own personal values. Dividing the perceived responsibility of acting by the number of witnesses can also impede helping behavior from taking place (see Fisher et al., 2011). Other variables that play an important role are the assessment outcomes of (non)intervening, the degree of victim blaming, how private the problem is considered, and the severity of violence (Fisher et al., 2011; Gracia et al., 2009). For instance, Gracia et al. (2018) found that people less prone to call the police were less willing to help an IPVAW victim. They considered the issue a private matter and showed higher rates of victim-blaming attitudes and hostile sexism.

Finally, community variables, such as collective efficacy, can also affect helping behavior among neighbors (Banyard et al., 2019). Collective efficacy refers to neighborhood residents' willingness and ability to act and resolve a community problem. It depends on social cohesion (sharing social norms and beliefs against IPVAW), social capital (the community's ability to prevent or intervene), and informal social control (action taken to try to tackle it) (Cowling, 2011). Research has linked collective efficacy to greater levels of bystander actions and lower levels of violence (see Banyard, et al., 2017). For instance, Browning (2002) found that neighborhoods with lower rates of lethal and nonlethal partner violence had higher levels of social cohesion and informal social control. Although other studies point to the contrary, this could be because a neighborhood's collective efficacy can be conditioned by the degree of social disorder (e.g., drug consumption/dealing, crime, or prostitution), creating attitudes of powerlessness in violent situations (Gracia & Herrero, 2007). In short, a large variety of variables seem to play a role in willingness to help IPVAW victims. Social cognition models such as the reasoned action approach (RAA: Fishbein & Ajzen, 2010), with substantial evidence of the prediction of multiple behaviors (Armitage & Conner, 2001), can collect their influence through a unique framework. This may facilitate the design of prevention campaigns aiming to promote helping behavior among neighbors.

**Figure 1.**
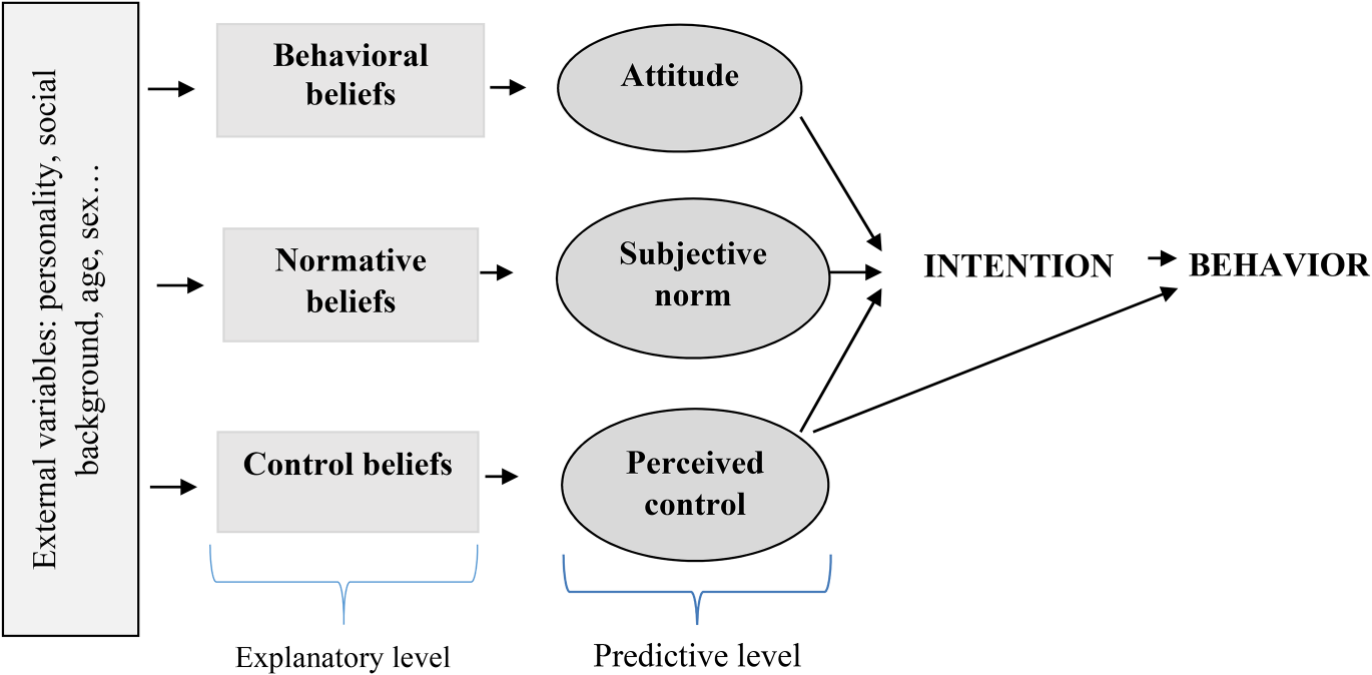
Schematic illustrating the reasoned action approach.

The RAA (Fishbein & Ajzen, 2010) states that a person's intention to help an IPVAW victim is a good motivational predictor of future behavior. In turn, attitudes, subjective norms, and perceived control are predictors of intention. A person's attitude towards helping the victim refers to an overall appraisal of the positive and negative consequences of carrying out the behavior. The subjective norm (or perceived social norm) includes two components: the injunctive norm, which is the perception of significant others approving or disapproving of one helping the victim, and the descriptive norm, which alludes to the perception of whether or not those significant others would help the victim. Perceived control refers to the degree to which a person perceives him/herself capable of helping the victim according to internal (i.e., skills) and external factors (i.e., effective resources). This last construct also has a direct effect on the performance of a behavior: People can have a strong intention to help a victim, but may not take action if in that precise moment he/she perceives low control (Figure 1). In addition, some authors state that past behavior can exert influence on intentions when behaviors have to take place in difficult contexts (Ouellette & Wood, 1998). In our case, having a previous positive or negative experience in reporting a case of IPVAW happening in one's neighborhood could affect actual intention. For this reason, together with the RAA constructs, we will analyze its contribution as a predictor of the intention to help. Identifying which variables are predictive of the helping behavior is crucial for designing effective social media campaigns. But how can we exert changes on these variables?

According to the RAA (Fishbein & Ajzen, 2010), the aforementioned predictive constructs are determined, respectively, by behavioral, normative, and control beliefs. Behavioral beliefs refer to the positive and negative outcomes of performing the behavior. They configure a person's attitude towards performing a behavior, depending on: (a) how positive or negative each consequence is for the person (outcome evaluation), and (b) how likely she/he perceives that each outcome will occur when reporting an IPVAW case to the authorities (behavioral belief strength). Normative beliefs allude to important prescriptive and descriptive referents. The influence of the prescriptive referents on a person's subjective norm depends on: (a) the degree to which each referent prescribes or proscribes their performing the behavior (injunctive belief strength), and (b) their motivation to comply with each one of them. Similarly, the influence of the descriptive referents on a person's perceived subjective norm depends on: (a) the degree to which each important referent performs or would perform the behavior (descriptive belief strength), and (b) the degree to which he/she self-identifies with each one of them. Finally, control beliefs refer to factors that people can perceive as facilitators and barriers to perform the behavior. Their impact on a person's perceived control depends on: (a) how likely he/she believes that the facilitators and barriers will be present when performing the behavior (control belief strength), and (b) the perceived power of each factor as a facilitator or inhibitor (power of control factors) (Figure 1). These beliefs in turn can collect the influence of other variables such as personal factors (e.g., sex, gender, personality traits), contextual factors (e.g., the degree of victim blaming, how private the problem is considered, the severity of violence), and community factors (e.g., social capital, neighborhood social disorder, collective efficacy). For instance, low levels of neighborhood social disorder may exert influence on people's intention to help by perceiving more prescribers and wanting to comply with them.

The identification of these beliefs is of extreme relevance as they can be used in social media campaigns to boost the construct(s) that best predict the helping behavior. Although there are divergent understandings on the extent and nature of media guidance in audiences, experts agree on their influence either in prolonging violence against women or confronting myths and stereotypes (Easteal et al., 2015). Governments and not-for-profit organizations (NGOs) have employed advertising, mass-media campaigns, educational kits, and community events as their primary methods to enhance communities' responsibilities towards IPVAW (Moreno-Martína et al., 2019). Initially, media campaign prevention initiatives have focused primarily on victims to make them aware of signs of abuse and of the existence of support services. Over time, they have also aimed at reframing IPVAW as a public issue to the general public and challenge attitudes and beliefs that normalize it (Lavack, 2010; Moreno-Martína et al., 2019). It is worth noting that some campaigns also target witnesses to precisely promote bystander interventions by confronting social norms, underlining bystanders' responsibility, denoting bystander capability and the need to intervene in IPVAW situations (Cismaru et al., 2014; Lavack, 2010). Although there is no evidence to support the effectiveness of social campaigns on IPVAW prevalence or incidence rates, in combination with other methods such as group training on gender equality, they are effective interventions (UN Women, 2016). According to experts, awareness campaigns are not theory-driven enough to change norms or actual behaviors, which results in a lack of theory incorporation to the design, implementation, and campaign evaluation (Heise, 2011; Lavack, 2010). For this reason, they recommend the use of evidence-based behavioral models (Heise, 2011). This, combined with the lack of formal research with a target audience, hinders judging those that are most effective (Lavack, 2010).

Prior to the pandemic, several media campaigns carried out in the United States, Australia, New Zealand, the United Kingdom, India, Spain, France, and South Africa aimed to encourage bystander intervention in IPVAW cases, including a call to action for neighbors (Family Violence Prevention Fun in the USA, 2003; see Lavack, 2010; Ministerio de Sanidad, Servicios Sociales e Igualdad, 2017; POWA, 2010; Secrétariat d’État de la Rèpublique Francaise, 2018). In line with other studies (Lavack, 2010), no information was found regarding their theoretical support nor regarding those launched during the pandemic (Femicidio.net, 2020; Instituto Andaluz de la Mujer, 2020). In most cases, they tackle attitudinal aspects and behavioral outcomes by calling for a spectator's social responsibility to act, reminding them that IPVAW is a social issue, or trying to anticipate their regret/guilt if the woman is killed. Indeed, attitudes towards IPVAW have been considered a central target to prevent this issue for their causal relationship to the perpetration of violence against women at an individual and community level (Flood & Pease, 2009). They reflect people's degree of acceptability regarding the use of violence in relationships and the communities’ response to the issue. In regards to the former, although studies on attitudes towards IPVAW are less frequent in high-income than in low-income countries (Gracia & Lila, 2015), there are disturbing findings. For instance, in Australia, one in five people believe that if the aggressor loses control or truly regrets what happened, excuses can be accepted. Two out of 10 people believe that men must take the lead in relationships and in the household, and three in 10 perceive that women want men to be in charge of relationships (Webster et al., 2018). In Europe, the largest review study (40 studies from 19 different countries) concluded that victim-blaming and sexist attitudes were still widespread and that specific violent behaviors were not only seen as tolerable but also inevitable (Gracia & Lila, 2015). In Spain, 7% of the population considers IPVAW as inevitable and even acceptable in certain circumstances (GDGV, 2014), and hard work with young people is still needed (Sánchez-Prada et al., 2020). Regarding how various sociodemographic variables affect attitude configuration, results are inconsistent: Either there are no differences or those found show low variance. Despite this, various studies have found that sex and gender, education, income level, and attitudes towards gender equality influence attitudes towards violence against women (see Webster et al., 2018).

Regarding how attitudes towards IPVAW affect victims’ friends, family and neighbors’ response to IPVAW, there is evidence that people with attitudes supporting the use of violence are more likely to blame the victims for the incidents and less likely to report it to the police (see Flood & Pease, 2009; Gracia et al., 2020). For instance, a multilevel analysis based on a European survey with more than 13,000 people showed that 32% of men knew an aggressor and found the use of violence against women more acceptable. This group of men were less likely to report an aggression, reducing the informal social control on IPVAW (Gracia & Herrero, 2006a). These same authors, in their study with a national probabilistic sample composed of nearly 2,500 Spanish citizens (2006b), found that those who held more positive attitudes towards reporting a case of IPVAW presented lower tolerance, perceived it as a frequent issue in society, and discussed it publicly. In contrast, 73.3% of participants with positive attitudes would not report (Gracia & Herrero, 2006b). This apparent inconsistency between attitude and behavior could be due to several factors including: (a) attitudes are contingent and contextual (Arriaga et al., 2016; Flood & Pease, 2009) and, (b) a person's attitude towards the target (IPVAW) does not necessarily coincide with people's attitudes towards themselves performing a specific behavior (reporting). In fact, such difference in the focus of study reflects the two traditions in social psychology in addressing the attitude–behavior relationship: target versus behavior (Eagly & Chaiken, 1998). Given that research on the attitude–target relationship is not always consistent with behavior (Pease & Flood, 2008; Webster et al., 2018), in the present study, together with analyzing the predictive capacity of people's attitudes towards themselves performing the helping behavior, we aim to study if general attitudes towards ending IPVAW is a predictor of the behavior.

More recently, there is research showing that attitude has an impact on behaviors by means of social norms (Fishbein & Ajzen, 2010), which is why the former should not be considered the only or even the main objective of prevention strategies (Pease & Flood, 2008). Attitudes reflect and reinforce norms, structures, and practices at all levels of society (Pease & Flood, 2008). According to Bem (2002), the most effective way to change attitudes is to modify behaviors by changing social norms, public policies, and practices, as human beings tend to “stick to the norm” to avoid social disapproval (Pease & Flood, 2008). Moreover, there is evidence that perceived norms exert as much influence on behavior as the structural norms of a community (Rimal & Lapinski, 2015). Changing the former is expected to be less expensive than changing the latter in terms of time and economic expense, and thus becomes more efficient in promoting bystander behaviors. Nonetheless, in this respect, several studies point out that perceived control also affects the probability of bystanders intervening. In other words, if people do not feel competent or perceive external barriers to intervene, it is less likely they will do so (Chabot et al., 2018; Exner & Cummings, 2011; Hoxmeier et al., 2017; Lavack, 2010). Consequently, perceived control is another key element to take into account in prevention campaigns.

To summarize, the relevance of attitudes, perceived norms, and perceived control in the context of studying IPVAW and bystander behaviors supports the adequacy of applying the RAA (Fishbein & Ajzen, 2010) to the helping behavior under study. According to the reviewed literature, one study applied the RAA to analyze if manipulating social norms affected people's intention to intervene in dating violence situations and whether these effects were mediated by the predictive constructs of the model (Lemay et al., 2019). Another study applied an adaptation of a previous version of the RAA to predict the intention of helping an abused female friend (Nabi et al., 2002). In the present study, we apply the latest version of the model, complying with the authors’ requirements to predict the intention of helping a neighbor who was an IPVAW victim during the pandemic. Our aims were: (a) to identify the predictors of the intention of reporting a case (or suspicion) of IPVAW happening in one's neighborhood and, (b) to identify the most important behavioral, normative, and/or control beliefs underlying the predictors of the behavior.

## Method

### Participants

A total of 360 people participated in the study. Convenience sampling was used. The final sample was composed of 352 people residing in Spain (*n*_women_ = 256, *n*_men_ = 95, and *n*_other_ = 1), 69% from the Valencian Community, after eliminating eight questionnaires belonging to people living abroad. The mean age was 34.2 years old (*SD* = 0.71). In total, 98.3% had a Spanish nationality. Almost 71% of the sample had a high level of education (studying at university, graduated, or had a postgraduate degree). At some point in their life, of the total, 36% had the suspicion that a neighbor was an IPVAW victim and 16.2% were certain about it. Among those who suspected or were sure of living “next door” to an IPVAW victim, 13.4% reported it to the authorities in the past. Almost 81% thought that the Spanish Law against IPVAW was between slightly and totally insufficient (*M* *=* 5.21; *SD* *=* 0.93), and 39% were active participants in the cause of ending IPVAW at a professional or NGO level (*n* = 138).

### Measures

The questionnaire consisted of two parts: The first one was composed of nine questions, and the second one of 24. In the first section, we asked for sociodemographic data (sex, age, education levels, nationality, and place of residence). We also asked if they had ever had a suspicion or the certainty that a neighbor was suffering from IPVAW (for instance, because what they heard was clear, they saw evident signs of aggression or they spoke with the woman in question). Next, we asked if they had ever reported it to authorities and if they had ever worked in IPVAW contexts or were IPVAW advocates (dichotomous responses). This last measure was used as the strongest indicator of general attitude, given that we can presume people who fight against IPVAW hold positive attitudes towards ending this problem. Based on Sanchez et al.'s (2020) study, we also asked for the extent to which they believed that the Spanish law to protect IPVAW victims was insufficient or excessive as a general attitude-related indicator of the entire sample (7-point bipolar scale: 1 = *totally agree*; 7 = *totally disagree*).

The second part of the questionnaire included reasoned action constructs. Following the RAA requirements (Fishbein & Ajzen, 2010), the questionnaire was built on the basis of a formative study. We identified people's modal salient beliefs regarding helping a neighbor who is a victim of IPVAW (Serrano, 2017) and tested the scales that showed good psychometric properties (Valverde, 2019). In the present study, we specified that the questionnaire was about reporting to the authorities a hypothetical case of IPVAW in their neighborhood during the pandemic lockdown. We defined “reporting” as notifying the authorities of the suspicion or occurrence of an IPVAW situation in their neighborhood.

All RAA variables scoring was the average scores on each of the 7-point scales used to measure them. The wording of the scales changed according to the content of the item. Examples of items and wording of the scales can be found in the questionnaire (see Supplemental annex 1).

#### Behavioral Intention

We considered four items to assess the intention of performing the helping behavior (α *=* 0.74). Higher scores reflected a stronger intention of performing it.

*Attitudes towards the behavior*. The scale was composed of 12 pairs of bipolar adjectives that measured instrumental and experiential aspects of the attitude towards performing the helping behavior (α *=* .89). For instance, participants’ had to answer on a 7-point scale whether they believed that reporting a case of IPVAW happening in their neighborhood was good/bad or useful/useless. Higher scores showed more favorable attitudes towards performing the behavior.
*Subjective norm*. We used six items, three to assess injunctive and descriptive norms, respectively (α *=* .78). Higher scores pointed to a higher perceived social pressure to perform the helping behavior.
*Perceived control*. We included three items to measure perceived control. Higher scores pointed to a higher perceived control over performing the helping behavior (α *=*.52).

The modal salient beliefs, identified in a previous study (Serrano, 2017; Valverde, 2019), are in Supplemental annex 1. They were all assessed according to the expectancy-value model on a 7-point scale (i.e.,: 1 = *totally disagree*; 7 = *totally agree*)

*Behavioral beliefs*. Participants answered how good or bad each of the seven behavioral outcomes were (belief strength) and how likely they thought each one would take place if they had to report to the authorities that a neighbor was suffering IPVAW (outcome probability). The product between belief strength and the outcome's probability of occurring mirrored the impact or relevance of the salient belief. Higher scores reflected the beliefs’ higher relevance.
*Normative beliefs*. Participants answered the degree to which they believed that the seven prescriptive referents listed would approve of them performing the helping behavior (belief strength) and the degree of motivation to comply with each one of them. Participants also answered in the same way regarding whether or not the four descriptive referents would help a victim (belief strength), and the degree of self-identification with each referent. The product between belief strength and motivation to comply (prescriptive beliefs) and belief strength and a person's identification (descriptive beliefs) reflected the impact of each prescriptive and descriptive belief, respectively. Higher scores showed the referents' higher relevance as prescribers or behavioral models.
*Control beliefs*. Participants answered to what degree the six different factors may facilitate or inhibit them from performing the behavior (belief strength) and the chances of each factor taking place (belief probability). The product between control belief and probability reflected the factor's power as a facilitator or inhibitor. Higher scores reflected the factors’ higher relevance as facilitators or inhibitors.

Note that belief scales include positive and negative consequences, favorable and nonfavorable prescriptive and descriptive referents, and facilitators as well as inhibitors. As a result, the reliability of the scales does not need to be calculated (Fishbein & Ajzen, 2010).

### Procedure

This project was born during the COVID-19 lockdown in March 2020. Mass media and some Spanish politicians raised awareness fthat victims were experiencing abuse while being trapped with their aggressors. At the same time some NGOs and regional governments launched campaigns such as “You can count on me” to promote bystander intervention among neighbors (Feminicidio.net). Such initiatives inspired the authors to identify the mechanisms underlying the behavior of reporting an IPVAW case, hoping to provide useful findings for the development of future campaigns based on behavioral models. The first author of this article has research experience on the field of IPVAW and the RAA model.

Upon obtaining the approval of the ethics committee of the Miguel Hernández University, we launched an online version of the questionnaire through different social networks, asking people to disseminate the questionnaire to all contacts within the legal age (18 years old). We also emailed about 80 Spanish universities’ Equality Units and town councils requesting their collaboration in helping spread the questionnaire among students and employees. Participants had to explicitly consent to participate by ticking a box. Questionnaires were anonymous and were administered during the Spanish lockdown (from April 26th to May 9th, 2020).

### Statistical Analysis

We employed the SPSS version 22 for all analyses. There were no missing data as we indicated to the online questionnaire design program that all questions were compulsory. We analyzed the relationship between constructs (Pearson's correlation) and between the impact of each belief and the corresponding predictive RAA construct. We also carried out a hierarchical multiple regression analysis by first introducing *attitude, subjective norm*, and *perceived control* as predictors of intention and then adding *having previously denounced* or *worked in IPVAW contexts/women's rights NGO* in the regression.

## Results

### Descriptive Analysis

Results of the descriptive analysis are shown in Table 1. Having reported a case in the past (*r* = .11; *p*≤* *.005) and having worked with IPVAW victims or for a women's rights NGO (*r* = .15; *p *≤* *.001) correlated with the intention of helping an IPVAW victim. The degree of sensitivity towards the issue also correlated with having worked with IPVAW victims or for a women's rights NGO (*r* = .15; *p *≤* *.001) but not with attitudes towards performing the behavior nor intention of performing it.

**Table 1.** Regression Analysis on Participants’ Intention to Report a Case of IPVAW Occurring in their Neighborhood.

Criterion	Predictors	*M* (*SD*)	*R* ^2^	*F*	*df*	β
Intention		6.39 (0.80)	.33	57.74 (0.000)	3, 34	
	Attitude	5.65 (0.74)				**.11**
	Subjective norm	5.64 (0.93)				**.29**
	Perceived control	5.60 (1.20)				**.31**
	Past behavior^a^	–	.35	37.01 (0.000)	5, 34	**.10**
	Working IPVAW^a^	–				.07

*Note*. Bold = significant predictor.

a dichotomous variables.

### Predictive Relationships

The RAA variables accounted for 33% of the intention of helping a neighbor victim of IPVAW. All three constructs were significant predictors of the intention, although perceived control showed the highest beta. Together with *past behavior experience* and *having worked with IPVAW victims or for a women's rights NGO*, the variables explained 35% of the intention's variance, although among these two, only past behavior showed a significant beta (β = .10, *p *≤* *.005) (Table 1).

### Target Beliefs

#### Behavioral Outcome Beliefs

All behavioral outcomes correlated with the attitude score, from *r* = −.22 (*p *≤* *.001) to *r* = .48 (*p *≤* *.001). *Helping protect the victim* (r= .48; p ≤ .001) together with *punishing the aggressor* (*r* = .46; *p *≤* *.001) and *feeling good about oneself* (*r* = .45; *p *≤* *.001) were the ones with higher correlations with participants’ attitude towards performing the behavior. The average strength of the behavioral beliefs was higher than 4 and the average of each of the perceived outcomes of performing the behavior was higher than 5 (Table 2).

**Table 2.** Participants’ Behavioral Beliefs and Their Correlation With Attitude Towards Reporting an IPVAW Case in Their Neighborhood.

Behavioral Beliefs	Outcome evaluation *M* (*SD*)	Belief strength *M* (*SD*)	*Attitude r*
The aggressor will be fairly punish	6.83 (0.51)	4.85 (1.52)	**.46**
The victim will be protected	6.93 (0.32)	5.01 (1.41)	**.48**
You are helping to end IPVAW	6.86 (0.43)	4.42 (1.76)	**.38**
You are a role model to others	6.75 (0.64)	5.47 (1.20)	**.43**
You could aggravate violence	6.06 (1.56)	4.61 (1.29)	**.33**
You would feel good	5.51 (1.34)	5.39 (1.33)	**.45**
Suffer retaliation on behalf of the aggressor	5.47 (1.24)	4.18 (1.38)	**−.22**

*Note.* Bold = significant mean correlations between the expectancy-value index of beliefs (product between outcome evaluation and belief strength) and attitude measure.

#### Normative Beliefs

All prescriptive referents correlated with the subjective norm scale, from *r* = .31 (*p *≤* *.001) to *r* = .47 (*p *≤* *.001). Their *couples* (*r* = .47; *p *≤* *.001), *family* (*r* = .45; *p *≤* *.001), and *friends* (*r* = .45; *p *≤* *.001) were the prescriptors with higher correlations with the subjective norm average score. The average strength of the prescriptive beliefs was higher than 5, and the average of the motivation to comply with each referent was higher than 4.5 (Table 4).

**Table 3.** Participants’ Control Beliefs and Their Correlation With Perceived Control on Reporting an IPVAW Case in Their Neighborhood.

Control beliefs	Belief probability *M* (*SD*)	Belief strength *M* (*SD*)	*Perceived control* *r*
To report anonymously and be protected during the process	6.42 (1.06)	4.40 (1.74)	**.11**
Have proof of the aggression to be an eyewitness	6.26 (1.14)	3.95 (1.65)	**.29**
To know that the victim would not want me to report	3.45 (1.58)	3.53 (1.46)	.04
To know that the victim will always be protected	6.80 (0.67)	4.60 (1.65)	**.25**
To know that the justice system and the police will act and effectively protect the victim	6.74 (0.76)	4.54 (1.68)	**.18**
That the people who are important to me will support me	5.87 (1.24)	5.77 (1.29)	.04

*Note.* Bold = significant mean correlations between the expectancy-value index of beliefs (product between belief probability and belief strength) and perceived control measure.

Regarding descriptive beliefs, significant correlations were found with the subjective norm score, from *r* = .35 (*p *≤* *.001) to *r* = .38 (*p *≤* *.001). The average strength of the descriptive beliefs was above 4.5 and the average score of participants’ self-identification with referents was above 3.9 (Table 3).

**Table 4.** Participants’ Normative Beliefs and Their Correlation With Subjective Norm With Respect to Reporting an IPVAW Case in Their Neighborhood.

Prescriptive beliefs	Motivation to comply *M* (*SD*)	Belief strength *M* (*SD*)	Subjective norm *r*
My family	5.35 (1.56)	5.85 (1.33)	**.45**
My friends	5.63 (1.34)	6.28 (1.01)	**.45**
My neighbors	4.65 (1.51)	5.05 (1.28)	**.36**
My couple	5.75 (1.36)	6.21 (1.24)	**.47**
Society	5.09 (1.41)	5.73 (1.04)	**.30**
My colleagues	5.27 (1.44)	6.05 (1.07)	.**31**
Descriptive beliefs	Self- identify * M* (*SD*)	Belief strength * M* (*SD*)	Subjective norm *r*
My friends	5.38 (1.46)	5.88 (1.08)	**.35**
My family	5.15 (1.59)	5.48 (1.47)	**.35**
My neighbors	3.94 (1.57)	4.60 (1.33)	**.38**
Society	3.97 (1.51)	4.71 (1.31)	**.35**

*Note*. Bold = significant mean correlations between the expectancy-value index of beliefs (product between motivation to comply or self-identify and belief strength) and subjective norm measure.

#### Control Beliefs

Not all of the factors showed significant correlations with the perceived control scale. Among those that did, correlations ranged from *r* = .11 (*p *≤* *.005) to *r* = .29 (*p *≤* *.001). The average strength of these beliefs was above 3.5 and their perceived power as a facilitator above 6. The facilitators with higher correlations with perceived control were *having evidence of the abuse or being a direct eyewitness* (*r* = .29; *p ≤ .001*) and *knowing that the victim will be protected* (*r* = .25; p ≤ .001) (Table 3).

## Discussion

The increasing global trend in the growth of IPVAW reports will continue during the pandemic; the known cases only represent the tip of the iceberg (Campbell, 2020). As social restrictive situations and confinements are still happening due to new outbreaks of COVID-19, we believe it is of the utmost importance to engage citizens in reporting IPVAW cases in their neighborhoods (van Gelder et al., 2020), especially given that in the present study half of the sample had at some point in their lives a suspicion or the certainty that a neighbor was a victim of IPVAW but the great majority did not report it. Similar results have been found in previous studies (European Commission, 2016). In our study, the participants’ sensitivity towards the issue could have made them more aware of it in their social circles, which is why many knew of or suspected a case. Yet, it seems this is not a sufficient condition to perform the helping behavior under study. In this regard, there is a potential likelihood that by intervening in the determinants of the intention to help a neighbor who was a victim of IPVAW, this would better promote the behavior among the population. To identify them, we applied the RAA (Fishbein & Ajzen, 2010).

The model explained moderate proportions of the intention of the sample to report to the authorities that a neighbor was a victim of IPVAW, which is in line with the average that has been established for intention in two meta-analytic studies (Armitage & Conner, 2001; McDermott et al., 2015). Perceived control was the most significant predictor, followed by the subjective norm and by attitudes. When adding to the regression *having worked with victims at a professional/NGO level* and *having reported a case in the past*, only the latter acted as a predictor that barely widened the intention's explained variance. Past experience provides relevant information on perceived control (Fishbein & Ajzen, 2010), and thus could strengthen behavioral facilitators or weaken inhibitors, increasing a person's intention of helping. Nonetheless, the predictive capacity of past behavior was weak, maybe because as the authors of the RAA affirm, past experience can affect intention by molding actual attitudes, subjective norms, and perceived control (Fishbein & Ajzen, 2010).

As stated, in our study perceived control is the most relevant target in enhancing an audience's intention to help an IPVAW victim. This result is in line with other studies (Chabot et al., 2018; Hoxmeier et al., 2017) that have found that perceived control (understood as external barriers and/or self-efficacy) predicted bystanders’ interventions in intimate partner violence and sexual assault situations. In fact, Exner and Cummings (2011) identified that sexual assault prevention strategies among college students should boost self-efficacy and lower barriers to stimulate bystander interventions. Keeping in mind that perceived control is not only a predictor of intention, but also a necessary condition for intention to transform into behavior, social media campaigns should aim to increase it. For this purpose, two control beliefs are most relevant for it to work: having evidence of the abuse or being a direct witness, and knowing that the victim will be protected. Both beliefs are perceived as good facilitators of the behavior, although the chances in which participants believe they would take place are not strong enough. The first belief needs to be weakened as a facilitator since the chances of having evidence of the aggression from one's home may actually not be high. In addition, the line between an interpersonal conflict and some specific situations of IPVAW can be blurred from the point of view of an outsider located at his/her own house. Therefore, campaigns should educate people on how to distinguish between an interpersonal conflict and an IPVAW episode. But above all, people must be reminded that: (a) reporting a suspected crime is simply being cautious and can prevent many unwanted consequences such as a person's death, and (b) neighbors are only responsible for alerting the authorities; the police are responsible for collecting for evidence. In the case of misinterpreting a situation, no evidence would be found against the partner and thus he would not be brought to trial. The pressure of reporting without having clear proof of the abuse should be taken off the “shoulders” of citizens to reduce cognitive dissonance in ambiguous situations. Providing information about the fact that people have the option to report a suspicion anonymously could also help. It was also perceived as a facilitator but with an unclear likelihood of it taking place. Regarding the second facilitator (knowing that the victim will be protected), campaigns should include information on the number of victims that have been attended by different resources and services and emphasize the coordination between them. In this way, we may increase the perceived chances that the facilitator will take place. Indirectly, this would help invigorate the belief that the protection system is effective, another perceived facilitator of performing the helping behavior. According to a previous study, the Spanish population believes that coordination must be improved in order to more effectively help women (GDGV, 2014).

Subjective norm is also an important predictor of helping a victim, a finding in line with other studies (Rimal & Lapinski, 2015). For instance, perceived approval of helping behaviors and the perception that others would help predicted the greatest intention of assisting in certain sexual assault and IPVAW situations (Austin et al., 2016; Baldry & Pagliaro, 2014; Hoxmeier et al., 2018). Evidence suggests that intervening on perceived norms can be an efficient way to produce behavioral changes (Jewkes, 2017), without needing to target other beliefs that are more laborious to change (Gidycz et al., 2011; McDonald & Crandall, 2015). To increase perceived social pressure, some specific referents should be targeted in campaigns. People participating in our study believed that their *couples*, *friends*, and *family* prescribed the helping behavior. Thus, media campaigns could focus on making these referents’ values even more accessible to their audience's mind. Family and friends, together with neighbors, also appear as descriptive referents, although in general terms people perceive that these referents are more prescribers than modeling agents. Therefore, a complementary strategy should be that news media organizations start to visualize bystander behaviors as more frequent among common citizens similar to our families, friends, and neighbors (not as “heroes” as they are often labeled), to strengthen descriptive norms and indirectly prescriptive norms (Cialdini, 2001). In Lemay et al.’s study (2019), positive descriptive norms not only molded better willingness to intervene in dating violence situations than injunctive norms, but also increased perceived control to intervene in dating violence situations. People can perceive that helping behaviors are socially desirable (prescriptive norm). However, they may not perceive helping a victim as an easy task because they lack referents, generating goal conflicts and negative emotions (Lemay et al., 2019). In this sense, fostering positive descriptive referents could be advantageous because they facilitate information processing, shorten decision-making processes (Cialdini, 2001), and increase perceived control.

Despite the fact that previously, challenging attitudes has been considered a standard tool in reducing IPVAW rates through communities’ responses (Flood & Pease, 2009; Gracia & Herrero, 2006a), in light of our results, attitudes towards performing the helping behavior should not be the main aim of social media campaigns. It could be thought that the moderate to high level of sensitivity of our sample towards the issue may explain why attitudes are not good predictors of the intention of helping, but these variables did not correlate. Gracia and Herrero (2006b) found that 73.3% of participants with positive attitudes would not report. Thus, although holding positive attitudes may be a necessary condition to report, it is not sufficient, since they do not seem to discriminate (as well as perceived control and subjective norms do) between those who would help a victim and those who would not. In fact, working with victims at the professional or NGO level (these participants showed higher levels of sensitivity towards the issue) is not a predictor of the behavior. Perhaps in past decades, favorable attitudes towards ending IPVAW had to be the focus (the first step to help a victim is perceiving that it is a problem that concerns you). But nowadays, 92% of the Spanish population firmly rejects IPVAW, according to one of the latest macrostudies (GDGV, 2014). Thus, to exert changes on a neighbor's willingness to help IPVAW victims, the next step seems to be providing tools to increase behavioral control and intensify perceived social pressure to help.

## Limitations of the Study

First, a common limitation of studies based on available samples is that the results cannot be generalized since the sample may not represent the general population. In our study, the percentage of participants with favorable attitudes towards ending IPVAW may be surprising but, as stated, the Spanish population is highly sensitive towards the issue (GDGV, 2014). Second, future studies should increase the sample size to include low intenders of performing the behavior and identify which beliefs campaigns directed at this population should target. Colloration is necessary to increase the sample size; of the approximately 80 institutions contacted to promote the online questionnaire, only two town councils (Elche and Pedreguer) and four universities’ Equality Units (Cordoba, Sevilla, Zaragoza and Oviedo) collaborated. Probably the work pressure exerted by the pandemic was the reason for such scarce cooperation. With greater participation, we could not only reach a larger sample, but also a more diverse one. Analyzing the model according to age, sociodemographic criteria, autonomous communities, and neighborhoods may reveal valuable information to design more specific campaigns at a more local level, together with divulgation action and training workshops as recommended (Fulu et al., 2014). Third, it would have been helpful to perform a prospective study. However, the difficulty in having online contact with the participants for whom we had no personal data made that impossible. In this case, measuring past behavior but closer in time could be an option for future studies to deepen knowledge on the intention's predictive capacity. Fourth, it would have been useful to control the social desirability effect as well as introduce an IPVAW attitude scale (instead of one question as an attitude-related indicator of the sample). Nonetheless, the questionnaire was anonymous and making the questionnaire longer could have been detrimental to participation rates and the questionnaire's validity. Fifth, it could be that people who work with IPVAW victims or fight for equality, despite holding positive attitudes towards ending this issue, would prefer to help a victim through other means rather than reporting it to the authorities. This possibility should be taken into consideration in future studies. Finally, the reliability of the perceived control scale suggests that it needs to be improved, potentially by including more items that better capture participants’ internal and external perceived control. Nevertheless, it should also be noted that perceived control refers to facilitating and inhibiting factors, some implying high control and others low control, which can result in an internal inconsistency (Fishbein & Ajzen, 2010). This fact could also be behind the mild correlations between control beliefs and perceived control.

## Conclusion and Practical Implications

During the pandemic lockdown, several initiatives aimed to promote citizens’ reporting of IPVAW cases in neighborhoods because of the increase in the number of IPVAW cases. However, experts insist on the need to design social media campaigns based on well-established theories of change (Heise, 2011). This study intends to contribute to the area with the following findings: (a) the RAA seems to be a useful behavioral model to predict the intention to help a neighbor victim of IPVAW; (b) we identified the most relevant beliefs and predictive constructs to target in campaigns to promote reporting IPVAW cases among neighbors--unexpectedly, subjective norm and perceived control (not attitudes) should be the core of social media campaigns to increase people's intention of helping (Fishbein & Ajzen, 2010);--and (c) the questionnaire can be used to replicate the study with a more diverse and larger sample in order to design more specific campaigns.
